# A new dawn for Parkinson’s disease: commentary on the emergence of stem cell-based therapies

**DOI:** 10.1186/s40035-026-00556-2

**Published:** 2026-04-29

**Authors:** Rosalie Elvira, Wai Hon Chooi, Hongyan Wang, Eng King Tan, Zhi Dong Zhou

**Affiliations:** 1 Department of Neurology, National Neuroscience Institute, 11 Jalan Tan Tock Seng, Singapore, 308433 Singapore; 2 Signature Research Program in Neuroscience and Behavioral Disorders, Duke-NUS Medical School, 8 College Road, Singapore, 169857 Singapore; 3 Department of Neurology, Singapore General Hospital, Outram Road, Singapore, 169608 Singapore

**Keywords:** Dopaminergic neurons, Clinical trials, Parkinson’s disease, Stem cell-based therapies

## Abstract

The field of regenerative medicine for Parkinson’s disease (PD) has reached a pivotal moment. After decades of preclinical research, recent first-in-human clinical trials demonstrated that cell replacement therapy using stem cell-derived dopaminergic neurons is not only feasible and safe but also shows promising signs of efficacy. Here we analyze three landmark 2025 studies, including the phase I/II trial of allogeneic induced pluripotent stem cell-derived dopaminergic progenitors, that mark a significant leap forward for PD therapy. We discuss principles underpinning the therapy, the historical context of fetal tissue transplants, findings from recent trials, and critical challenges. The convergence of robust cell manufacturing, precise stereotactic surgery, and advanced neuroimaging provides compelling evidence that stem cell-based therapies are potentially a viable treatment paradigm for PD.

## Introduction

Parkinson’s disease (PD) is the second most common neurodegenerative disease, characterized by a progressive loss of dopaminergic neurons in the substantia nigra pars compacta. This specific cell loss leads to severe depletion of the neurotransmitter dopamine (DA) in the striatum, manifesting clinically as classic motor symptoms including tremor, bradykinesia, rigidity, and postural instability. For over half a century, the mainstay of treatment has been pharmacological DA replacement, primarily with levodopa (L-DOPA) [1]. While highly effective initially, long-term use of L-DOPA is complicated by motor fluctuations and dyskinesia. Deep brain stimulation (DBS) provides significant symptomatic relief for selected patients but does not halt the underlying progressive neurodegeneration [2].

As a well-defined pathology of PD, the loss of a single and specific neuronal population has long made PD an ideal candidate for cell replacement therapy. The fundamental hypothesis is simple: replacing the degenerating DA-producing neurons to restore the tonic DA release and re-establish normal circuits in the striatum. Early proof-of-concept came from transplantation of fetal ventral mesencephalic tissue, which contained the necessary dopaminergic progenitors [3, 4]. These open-label studies showed that such grafts could survive for decades and provide long-term motor benefits. However, the use of fetal tissue was plagued by ethical concerns, limited tissue availability, and variable cellular compositions, which contributed to inconsistent outcomes and a high incidence of graft-induced dyskinesias (GIDs) in subsequent randomized controlled trials [5, 6].

The discovery of human pluripotent stem cells (hPSCs), including both human embryonic stem cells (hESCs) and induced pluripotent stem cells (iPSCs), is a promising solution. These cells offer a theoretically limitless, standardized, and ethically sound source for generating the required dopaminergic neurons. After years of refining differentiation protocols, the recent publication of the first phase I/II trial of allogeneic iPSC-derived dopaminergic progenitors in *Nature* signals that stem cell therapy for PD is transitioning from a distant dream to a tangible reality [7–9]. This trial, alongside others, provides critical data on the safety, feasibility, and early efficacy, marking a new chapter in restorative neurology.

To provide a focused analysis of the most advanced clinical data on the safety and feasibility of this approach, we selected three landmark studies published in 2025 that reported safety and efficacy outcomes after 12 to 24 months of treatment: the Phase I/II trial of allogeneic iPSC-derived dopaminergic cells (Sawamoto et al.) [7], and the Phase I trials of hESC-derived dopaminergic neurons (Tabar et al.) [8] and hESC-derived DA progenitors (Chang et al.) [9]. Other important ongoing trials, such as the STEM-PD study (Novo Nordisk) [10], have reported protocols and interim safety communications, but their primary outcomes have not been peer-reviewed and thus fall out of the scope of this commentary.

## Previous achievements: lessons from fetal tissue transplants

The modern era of cell therapy is built upon the foundational evidence from fetal tissue transplantation. Initial open-label studies offered the first definitive proof that the grafted dopaminergic neurons could survive long-term in the human brain, integrate into the host circuitry, and provide sustained clinical benefits [11]. However, subsequent double-blind, placebo-controlled trials failed to meet their primary endpoints and revealed a high incidence of GIDs [12]. These early challenges were not due to the failures of the concept itself, but rather reflected the limitations inherent in using primary fetal tissue.

The most critical lesson learned was that cell purity is paramount for clinical safety. Fetal tissue grafts were heterogeneous; subsequent retrospective research suggested that contamination with serotonergic neurons within those grafts may contribute to the development of GIDs [13]. In contrast, modern stem cell protocols prioritize the generation of high-purity, standardized dopaminergic progenitors to eliminate such off-target effects [9, 14, 15]. Furthermore, these early trials underscored the importance of patient selection, as subgroup analyses suggested that younger patients with less advanced disease stages were significantly more likely to benefit from the cell therapy. Finally, the inconsistent outcomes from fetal tissue transplantation highlighted that robust, standardized immunosuppression is a necessity rather than an option. Inconsistent or inadequate regimens in the past likely led to silent graft rejection. Current protocols address this problem through strategic use of human leukocyte antigen (HLA)-matched lines and disciplined pharmacological regimens like tacrolimus. This inconsistent outcome was attributed not only to the presence of contaminating serotonergic neurons within the grafts [13] but also to the uneven, ‘patchy’ re-innervation of the putamen, which created localized dopamine hotspots [5]. Modern stem cell protocols and surgical delivery techniques aim to address both of these issues by prioritizing high-purity dopaminergic progenitors and utilizing multi-track injection paradigms to achieve more homogeneous graft coverage.

## Principles of stem cell therapy for PD

The goal of cell therapy in PD is to reconstruct the damaged neural circuitry by transplanting cells that can mature into authentic, functional A9-type midbrain DA neurons, which is the very population lost in PD. These A9 DA neurons specifically project to the dorsal putamen, the motor compartment of the striatum [16].

### Molecular orchestration of dopaminergic fate

The transition from hPSCs to specialized A9-type neurons is a sophisticated bioengineering feat that relies on the precise recapitulation of embryonic signaling. Because hPSCs possess the innate capacity to differentiate into any cell lineage, they must be strictly sequestered into a neural fate through a process known as dual-SMAD (suppressor of mother against decapentaplegic) protein inhibition [14, 17]. This initial step blocks alternative pathways, such as bone morphogenetic protein (BMP) signaling, effectively “canalizing” the cells toward a neuroectodermal identity. Once this neural commitment is established, the cells must be assigned a specific regional identity.

To achieve a midbrain dopaminergic phenotype, researchers meticulously manipulate the sonic hedgehog (SHH) and Wnt signaling pathways using potent small molecules. The activation of SHH provides the necessary “ventralizing” signal, while the temporal control of Wnt signaling, often via GSK3 inhibitors, provides the “positional” instructions that specify a midbrain, rather than a forebrain or hindbrain, identity [14]. A critical milestone in this journey is the induction of a floor-plate intermediate stage. This stage is marked by the expression of key transcription factors, including LIM homeobox transcription factor 1α (LMX1A) and Forkhead box A2 (FOXA2), which serve as biological signatures of a floor-plate intermediate. To precisely specify a caudal midbrain identity, additional markers are required. The co-expression of Orthodenticle homeobox 2 (OTX2) and Engrailed-1 (EN1) with FOXA2 and LMX1A is critical for distinguishing authentic A9 dopaminergic progenitors from those of diencephalic or subthalamic origin. [13, 14].

Following the induction of this progenitor state, the focus shifts to the post-transplantation phase, where the cells must complete their maturation within the host environment. This “in vivo” transition is a long process occurring over several months [18]. During this period, the progenitors must not only survive the mechanical trauma of stereotactic delivery but also undergo terminal differentiation into mature, DA-secreting neurons. Success is defined by the cells’ ability to achieve synaptogenesis, potentially integrating with the existing host circuitry, with the goal of establishing a system for physiologically regulated release of DA. However, it remains to be determined whether the transplanted neurons achieve full synaptic integration or function primarily as a tonic source of dopamine release, which is a critical distinction for long-term physiological regulation.

### Putamen as the therapeutic target

The clinical efficacy of stem cell-derived therapy in PD is predicated based on the anatomical accuracy of the graft delivery. The primary surgical target is the putamen, specifically the dorsal region, which functions as the principal motor compartment of the striatum [19]. By bypassing the degenerated substantia nigra and transplanting cells directly into the putamen, the therapy seeks to achieve “ectopic” re-innervation, placing the DA-producing machinery exactly where it is needed to restore functional motor loops [19, 20].

The surgical delivery of these progenitors via stereotactic neurosurgery represents a shift from simple injection to precise anatomical reconstruction [21]. By utilizing multiple needle trajectories and depositing ‘boutons’ of cells along each track, researchers ensure broad, homogeneous re-innervation of the motor striatum [22]. This spatial distribution is vital for promoting functional graft–host integration and avoiding the ‘patchy’ DA levels that historically contributed to inconsistent therapeutic gains [21]. Furthermore, the transition to specialized micro-cannulas serves a dual purpose: it minimizes mechanical trauma to the host tissue and reduces the localized inflammatory milieu, thereby enhancing the survival and maturation of the transplanted progenitors in a potentially hostile Parkinsonian environment [23].

A significant evolution in this therapeutic strategy is the transition toward allogeneic, “off-the-shelf” iPSC products. While autologous (patient-specific) transplants eliminate the need for immunosuppression, the logistical and financial hurdles of manufacturing individualized cell lines are prohibitive for large-scale clinical adoption. The strategic use of pre-validated, high-quality donor cell lines, such as the HLA homozygous donor line, which was selected for a common HLA haplotype present in approximately 17% of the Japanese population utilized in the Kyoto University trials [7], represents a paradigm shift toward scalability. This approach allows for rigorous quality control and standardization of the cell dose, ensuring that the final product delivered to the putamen is consistent in potency, purity, and safety across a wide patient population.

### The immune challenge

The shift toward allogeneic “off-the-shelf” products introduces a significant immunological hurdle: the potential for graft rejection [24]. Unlike autologous transplants, allogeneic stem cell-derived neurons are recognized as foreign by the host’s immune system [25, 26]. While the central nervous system possesses a degree of immune privilege, the disruption of the blood–brain barrier during stereotactic surgery, combined with the presence of foreign HLA markers on the transplanted cells, can trigger a robust T-cell-mediated response [27]. To mitigate this risk, patients must undergo a rigorous immunosuppressive regimen, typically modelled after protocols used in solid organ transplantation [28].

In contemporary clinical trials, such as the iPSC study at Kyoto University, the macrolide immunosuppressant tacrolimus has been the primary agent of choice [7]. Administered for a duration of approximately 15 months, tacrolimus functions by inhibiting calcineurin, thereby preventing the activation of T-lymphocytes that would otherwise target and destroy the grafted progenitors. A key strategic refinement in these trials is the use of a donor line for a common HLA haplotype, aiming to reduce the “immunological distance” for a subset of matched recipients. However, the clinical data from this trial showed no overt difference in safety or efficacy between HLA-matched and -unmatched patients [7], suggesting that the role of HLA-matching in this context remains to be fully defined.

Despite these precautions, the long-term immunological fate of the graft remains the most significant “unanswered question” in the field. The current clinical standard is to withdraw immunosuppression after the initial maturation phase (roughly one to one and a half years post-surgery), under the hypothesis that the blood–brain barrier will have repaired itself and the mature neurons will have integrated sufficiently to survive [7, 28]. However, the risk of chronic, low-grade inflammation or delayed rejection persists [29]. Future research is now pivoting toward the development of “immune-evasive” or “universal” donor cells, engineered via the clustered regularly interspaced short palindromic repeats (CRISPR)/CRISPR-associated protein 9 (Cas9) protocol to lack MHC molecules, which could eventually eliminate the need for systemic immunosuppression [30]. Altogether, this marks the final step toward a truly accessible regenerative therapy for PD.

## Analysis of the recent iPSCs trial: a landmark for regenerative medicine

**Fig. 1:**
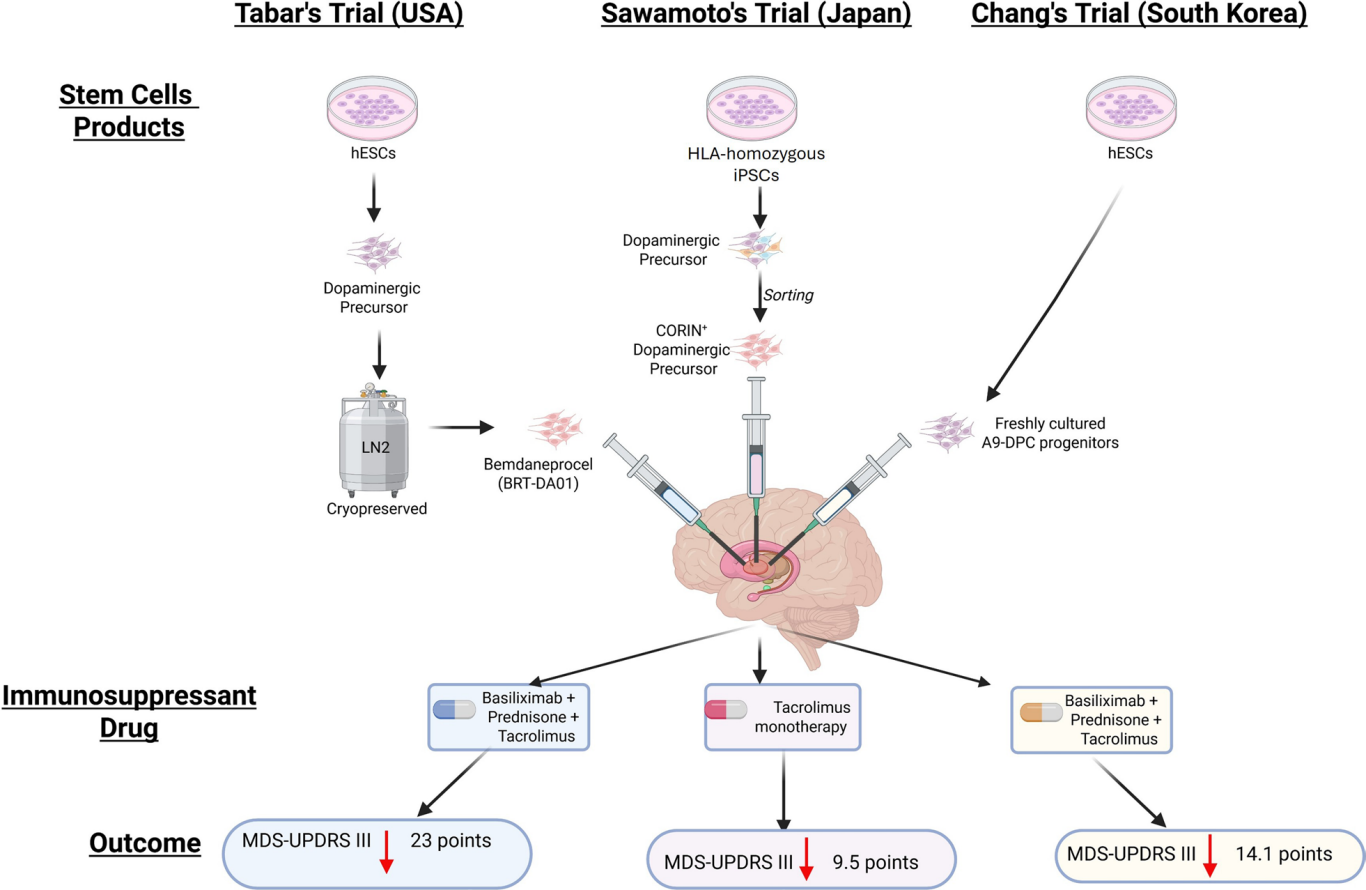
Comparative overview of the three first-in-human clinical trials for PD using stem cell-derived dopaminergic precursors. Key features of three landmark 2025 trials were summarized: the Sawamoto’s Trial in Japan, the Tabar’s Trial in USA/Canada, and the Chang’s Trial in South Korea. The three trials employed distinct stem cell sources and manufacturing strategies: the HLA-homozygous iPSCs (CORIN + sorted), the cryopreserved hESC-derived progenitors (Bemdaneprocel), and the freshly cultured hESC-derived A9-DPCs, respectively. Immunosuppressive regimens varied, with Sawamoto’s trial using tacrolimus monotherapy and the other two trials adopting triple-drug protocols. Motor outcomes were assessed via MDS-UPDRS Part III scores in the “OFF” state, revealing dose-dependent improvements: − 9.5 (Japan), − 23 (USA), and − 14.1 (Korea). These results collectively demonstrate the safety, feasibility, and early efficacy of stem cell-based cell replacement therapy for PD. Figure created with BioRender.com

The year 2025 represents a milestone in restorative neurology, marked by the nearly simultaneous publication of results from three geographically and technically diverse trials. Collectively, these studies establish safety as a foundational milestone for stem cell therapy in PD (Fig. 1). To provide a cohesive narrative of these three landmark 2025 trials, in the following, we summarize the trial designs, safety outcomes, and efficacy signals for each study (Table 1).

**Table 1:** Summary of clinical trials of hPSCs used in PD therapy

Trial & publication	Stem cell source	Product/strategy	Patients & doses	Immunosuppression	Key safety findings	Motor outcomes	Imaging evidence	Notable features
Sawamoto et al., *Nature* May 2025 (Kyoto, Japan) [7]	iPSCs (allogeneic, HLA-homozygous)	CORIN^+^ sorted dopaminergic progenitors	7 patients (low dose: ~ 2.5 million cells; high dose: ~ 5.5 million cells per hemisphere)	Tacrolimus monotherapy, tapered at 12 months, stopped at 15 months	No serious adverse effects; 73 mild/moderate events; no tumors or graft-induced dyskinesia	OFF score: mean − 9.5 points; ON score: mean − 4.3 points; Hoehn-Yahr improved in 4 patients	PET: ↑ 44.7% dopamine synthesis overall; high-dose group ↑ 63.5%	First robust clinical validation of allogeneic iPS therapy; scalable “off-the-shelf” model
Tabar et al., *Nature* July 2025 (MSK/BlueRock, US/Canada) [8]	hESCs	Cryopreserved progenitors (bemdaneprocel, BRT-DA01)	12 patients (low dose: 0.9 million cells; high dose: 2.7 million cells per putamen)	Basiliximab + prednisone + tacrolimus for 1 year, then stopped	No graft-related adverse effects; no tumors; no graft-induced dyskinesia	High-dose cohort: mean − 23 points in OFF MDS-UPDRS; + 2.7 h/day “Good ON” time	PET: ↑ ^18^F-DOPA uptake confirming graft survival	Precision MRI-guided delivery; durable benefit sustained at 18 months post-transplant
Chang et al., *Cell* Dec 2025 (Korea) [9]	hESCs	Freshly cultured A9-DPC progenitors	12 patients (low dose: 3.15 million cells; high dose: 6.3 million cells)	Basiliximab + prednisone + tacrolimus for 12 months	32 adverse effects, none graft-related; 1 serious adverse effect (thrombocytopenia, resolved); no tumors	OFF score: mean − 14.1 points overall; Hoehn-Yahr improved (− 1.0 low, − 1.7 high)	PET: ↑ dopamine transporter binding, especially in high-dose group	Fresh (not cryopreserved) product; dose-dependent efficacy; interim 12-month analysis

### Phase I/II trial of iPSC-derived dopaminergic cells

This landmark study utilized fresh dopaminergic progenitors derived from a clinical-grade iPSC cell line [7]. To ensure scalability and minimize rejection, the researchers used an allogeneic cell source from a healthy donor homozygous for a common HLA haplotype, matching approximately 17% of the Japanese population. The trial employed a dose-escalation design where three patients received a low dose (2.1–2.6 × 10^6^ cells/hemisphere) and four received a high dose (5.3–5.5 × 10^6^ cells/hemisphere). Patients were maintained on tacrolimus for 15 months to support graft integration. Importantly, while the cell line was selected for a common HLA haplotype, patients in the trial included both those who were HLA-matched and -unmatched to the donor line.

The trial reported an overwhelmingly positive safety profile over 24 months, with no graft-related serious adverse events, such as deaths, tumors, or graft overgrowth. This was confirmed via serial magnetic resonance imaging and the absence of proliferative signals on ^18^F-FLT positron emission tomography (PET) scans. Crucially, no GIDs were reported, an achievement attributed to the high purity of CORIN (a floor plate marker)-positive progenitors and the exclusion of serotonergic neurons. Encouraging efficacy signals were also observed; four of six evaluated patients showed improvements in the “OFF-medication” state (Movement Disorder Society–Unified Parkinson’s Disease Rating Scale (MDS-UPDRS) Part III) with an average improvement of 9.5 points (20.4%). Objective evidence of graft function was provided by ^18^F-L-DOPA PET imaging, which revealed a striking dose-dependent effect: the high-dose group showed a 63.5% increase in dopamine synthesis compared to 7.0% in the low-dose group.

### Phase I trial of hESCs-derived dopaminergic neurons

The BlueRock Therapeutics trial (investigating bemdaneprocel, often referred to as the ‘Tabar trial’ after the lead surgeon) utilized dopaminergic neuron precursors derived from hESCs [8]. This multi-center, open-label study enrolled 12 participants divided into two cohorts: a low-dose group receiving 0.9 million cells per putamen and a high-dose group receiving 2.7 million cells per putamen. Similar to the iPSC trial, participants received one year of immunosuppression to facilitate engraftment.

The primary objective of demonstrating safety and tolerability was met, with no serious adverse events related to the cell product reported through the follow-up period. Serial imaging and clinical assessments confirmed the absence of tumors or GIDs. Exploratory efficacy data were highly promising, particularly in the high-dose cohort, which showed a mean reduction of 23.0 points in the MDS-UPDRS Part III “OFF” score at one year. Motor benefits were sustained at 18 months after grafting, with continued improvement observed in the high-dose group after immunosuppression withdrawal [8]. PET imaging corroborated these clinical findings, showing sustained evidence of cell survival and functional engraftment.

### Phase 1/2a clinical trial of hESC-derived DA progenitors

This single-center, dose-escalation trial in South Korea evaluated high-purity A9-type dopaminergic progenitors (TED-A9) also derived from hESCs [9]. The study enrolled 12 patients with moderate-to-severe PD who received bilateral putaminal transplants. The cohorts were split between a low-dose group (3.15 million cells) and a high-dose group (6.30 million cells), with all participants receiving a standardized one-year immunosuppression regimen.

The safety results were consistent with the other landmark trials, showing no dose-limiting toxicities, no graft-related serious adverse events, and no evidence of tumor formation or overgrowth. At the 12-month mark, the trial reported significant motor improvements, with a mean 14.1-point reduction in MDS-UPDRS Part III “OFF” scores and greater benefit observed in the high-dose cohort. Although more modest thanthe 23-point reduction reported in the Tabar trial at 18 months, this still represents one of the most substantial improvements seen across the three studies. This clinical recovery was supported by ^18^F-FP-CIT PET imaging, which showed significantly increased DA transporter uptake in the posterior putamen, providing objective confirmation of successful graft survival and re-innervation. These findings further solidify the dose-dependent relationship between the number of transplanted progenitors and the magnitude of clinical improvement.

### Challenges of graft composition and functional yield

A critical yet often underappreciated aspect of these trials is the low final yield of authentic dopaminergic neurons within the grafts after transplantation. While significant effort is devoted to generating high-purity dopaminergic progenitors in vitro, the in vivo maturation process results in a substantially lower proportion of functional tyrosine hydroxylase (TH)-positive neurons. Preclinical data supporting these trials revealed this fundamental limitation. In the Kyoto trial, while single‑cell RT‑qPCR indicated ~ 60% of the transplanted cells were DA progenitors, post‑mortem analysis in rats showed that only 2%–5% of surviving human cells were TH^+^ dopaminergic neurons [7]. The hESC‑derived product in the Tabar trial yielded only ~ 9%–16% TH^+^ neurons in rat grafts [8]. Similarly, the South Korean trial reported 14.9% ± 12.8% of surviving human cells becoming TH-positive at 24 weeks post‑transplantation in rats [9].

This discrepancy suggests that the vast majority (≥ 85%) of transplanted cells differentiate into non‑dopaminergic cell types in vivo, including potentially glial or other neuronal lineages. This is not merely a survival issue but reflects the inherent heterogeneity of the progenitor population. Markers like FOXA2 and LMX1A, while indicative of floor plate identity, do not precisely define the caudal midbrain dopaminergic fate and can also label subthalamic and diencephalic populations [31]. This underscores that the current generation of cell products, while safe and effective, is likely operating at a fraction of their potential efficiency. Future advances will depend on the development of more precise differentiation protocols, such as quadruple FACS sorting (FOXA2^+^LMX1A^+^OTX2^+^EN1^+^) [31] or optimized protocols like the ‘Wnt boost’ method [32], retinoic acid (RA)-based caudalization [33], or other recently optimized protocols [34] to generate a purer starting population of midbrain dopaminergic progenitors, thereby maximizing the functional neuronal yield per transplanted cell.

A critical safety concern from the fetal transplant era, GID, was notably absent across all three trials. This is a significant milestone, likely attributable to the combination of high-purity dopaminergic progenitors (reducing serotonergic contamination) and the use of multi-track surgical delivery to promote more homogeneous graft innervation. However, continued long-term surveillance remains essential, as GID in historical cohorts sometimes emerged years after transplantation.

Taken together, these three clinical trials converge on a set of consensus points: stem cell-derived dopaminergic progenitors are safe, and can survive in the host brain, produce DA, and improve motor symptoms in a dose-dependent manner. Despite differences in stem cell source, manufacturing approach, and geographic setting, the results collectively mark a turning point in the field, overcoming the historical limitations of fetal tissue grafts and paving the way for larger, controlled studies that will define the long-term efficacy and durability of this therapeutic strategy.

## The road to standard of care: critical limitations and future hurdles

While the initial data from these landmark trials are promising, a rigorous evaluation requires a cautious interpretation, as several inherent limitations remain. A primary concern is the open-label design of these studies; the lack of a sham-surgery control group means that the observed improvements could be influenced by substantial placebo effects, which are common in neurosurgical interventions for PD. Furthermore, the small sample sizes mean that these studies were not statistically powered to prove clinical efficacy, serving instead to establish foundational safety. Notably, the degree of clinical improvement even for these open-label studies is currently not better than existing drug and surgery therapies. For example, established interventions such as DBS of the subthalamic nucleus have demonstrated mean improvements in the MDS-UPDRS Part III ‘OFF’ score of approximately 20–30 points in controlled trials, representing a well-established benchmark for surgical efficacy. The improvements reported in these stem cell trials, while promising and durable, have yet to definitively surpass this standard. Moving forward, the most immediate hurdle is the requirement for large-scale, double-blind, sham-surgery-controlled Phase III trials to truly validate these outcomes against the placebo effect.

Beyond trial design, the long-term risks associated with the immunosuppression burden remain non-trivial. While 12-to-15-month regimens of agents like tacrolimus appeared manageable during the initial maturation phase, the long-term immunological fate of allogeneic cells after drug withdrawal is still an “unanswered question” in the field. This logistical burden highlights the ongoing debate between allogeneic “off-the-shelf” products, which offer scalability but require drugs, and autologous approaches using a patient’s own cells, which are financially complex but potentially avoid immune rejection entirely. However, autologous transplants may not remove the underlying genetic predisposition of the patient.

Additionally, there are significant questions regarding the durability and disease progression, as a 24-month follow-up is relatively short for a chronic condition. The long-term safety of iPSC-derived progenitors and differentiated neurons remains a major concern. It remains unknown whether the transplanted neurons will provide lifelong benefit or eventually succumb to the underlying pathology of the host brain, such as the accumulation of α-synuclein (α-syn) protein. Furthermore, the functional modality of the graft, whether it achieves true synaptic integration with host circuitry or acts as a tonic dopamine ‘pump’, remains an unresolved question that will influence the long-term stability and physiological regulation of the therapeutic effect. Beyond optimizing the cell product itself, adjunctive pharmacological strategies may also enhance graft survival and function. For instance, preclinical and clinical data suggest that the anti-epileptic drug zonisamide, which has neuroprotective properties, could potentially improve the survival of transplanted dopaminergic neurons, a concept worthy of future investigation [35]. To address this, researchers must continue optimizing protocols to identify the ideal therapeutic window, as the optimal sites and number of grafted cells are still unclear, necessitating convergence on the most effective cell dose and precise transplantation targets.

Early data suggest that younger patients at less advanced stages of the disease may derive the greatest benefit; thus, identifying specific biomarkers for the “best candidates” will be critical for future success [7, 36]. However, the selection of candidates remains a major challenge because PD is heterogeneous, and host factors and the microenvironment significantly affect the graft-host integration.

Finally, the industry faces challenges in standardization and scaling. Transitioning to widespread application requires the development of industrial-scale, highly reproducible cell manufacturing processes conducted under rigorous GMP (Good Manufacturing Practice) conditions. However, standardization must extend beyond the lab to the operating room; current imaging often reveals focal “hotspots” of DA uptake rather than a broad, homogeneous restoration. Addressing this requires the standardization of surgical delivery hardware and multi-track protocols to overcome the anatomical challenges of re-innervation. These standardized systems are critical not only for consistent efficacy but also for practical scaling across clinical centers. Achieving full functional recovery will likely require the integration of higher cell doses, optimized delivery techniques, and refined patient selection.

## Perspectives

The future of stem cell-based therapies for PD is characterized by a blend of clinical excitement and the necessity for rigorous scientific inquiry to overcome remaining biological hurdles. One of the most significant technical hurdles is the re-innervation challenge. Current PET imaging often reveals focal “hotspots” of DA uptake at the transplant sites rather than the broad, homogeneous re-innervation required for full motor recovery. To address this, future research must explore the use of higher cell doses or more sophisticated delivery techniques, such as multi-track stereotactic injections, to establish the currently unclear optimal sites and number of grafted cells and ensure that the transplanted neurons provide more comprehensive coverage across the striatum. Ultimately, future trials must aim for a degree of clinical improvement that definitively surpasses current drug and surgery therapies, moving beyond the results seen in early open-label studies.

Furthermore, the long-term success of these grafts likely depends on the development of combination therapies. Because cell replacement only addresses the loss of DA, it must be paired with neuroprotective agents and anti-α-syn therapies to protect new neurons from the underlying disease process that continues in the host brain. This is critical because autologous transplants may not remove the patient’s underlying genetic predisposition, and there is a high risk of cell–cell propagation of α-syn pathology into the newly grafted neurons. Integrating these biological treatments with specialized physical rehabilitation may also be essential for functional incorporation of the new neural circuits and addressing the non-dopaminergic symptoms that cell therapy alone cannot fix.

Finally, the field is looking toward advanced cell engineering to simplify the treatment process and improve safety. A major challenge remains to be the selection of candidates; because PD is highly heterogeneous, future protocols must identify how specific host factors and the brain microenvironment affect graft-host integration. By leveraging gene-editing technologies like CRISPR/Cas9, scientists are working to create “hypoimmunogenic” or universal donor cells. These engineered cells could be “hidden” from the host’s immune system, potentially eliminating the need for long-term systemic immunosuppression. Most importantly, establishing the long-term safety of iPSC-derived progenitors and differentiated neurons remains a primary concern that must be resolved to make restorative therapy a much more accessible and manageable option for the millions of people living with PD.

## Conclusion

The recent success of pioneering trials like the Kyoto University study using allogeneic iPSC-derived dopaminergic progenitors is a landmark event in the history of neuroscience and regenerative medicine. These studies provide the first robust, peer-reviewed evidence that cell replacement therapy for PD is a safe and feasible intervention [7–9]. Additional ongoing clinical trials, such as the ASPIRO trial (NCT06344026), which explores the use of autologous iPSCs to eliminate the need for immunosuppression [37], and the STEM-PD trial (NCT05635409) in Europe, which is currently testing escalating doses to determine the minimal therapeutic threshold [10], will further inform the field. Tumorigenicity and graft-induced dyskinesias are absent, while the dose-dependent motor improvements and objective imaging evidence of graft function suggest a therapeutic effect. However, the results should be interpreted with caution, as the degree of clinical improvement observed in these early open-label studies has not surpassed the efficacy of current pharmacological and surgical standards. Furthermore, establishing the long-term safety of iPSC-derived progenitors and differentiated neurons remains a major concern that must be resolved to ensure the viability of this approach.

While the data collectively represent a paradigm shift, the transition to standard care faces significant biological and technical hurdles. The optimal transplantation sites and the precise number of grafted cells required for full recovery remain unclear. Furthermore, the low yield of functional dopaminergic neurons from the transplanted progenitors (often < 15% of surviving graft) represents a major inefficiency that must be addressed through next-generation differentiation and sorting protocols to maximize the therapeutic efficacy. Additionally, the selection of appropriate candidates is a major challenge because PD is highly heterogeneous, and individual host factors and the brain microenvironment may significantly influence the graft-host integration. It is also vital to consider that autologous transplants may not remove a patient’s underlying genetic predisposition, and there remains a high risk of cell-to-cell propagation of α-syn pathology from the host brain into the newly grafted neurons. These challenges move the central question from “Is this possible?” to “How can we make this better and available for all?”. The path forward requires larger, blinded trials, protocol optimization, and long-term monitoring. Nevertheless, for the millions of people living with PD, this trial heralds a new and hopeful dawn, a future where replacing the cells lost to neurodegeneration is not just a concept, but a concrete and potent therapeutic strategy. The dream of restorative therapy for PD has never been closer to reality.

## Data Availability

No datasets were generated or analysed during the current study.

## Abbreviations

α-syn: α-Synuclein
BMP: Bone morphogenetic protein
Cas9: CRISPR associated protein 9
CRISPR: Clustered regularly interspaced short palindromic repeats
DA: Dopamine
DBS: Deep brain stimulation
FOXA2: Forkhead box A2
GIDs: Graft-induced dyskinesias
hESCs: Human embryonic stem cells
hPSCs: Human pluripotent stem cells
iPSCs: Induced pluripotent stem cells
L-DOPA: Levodopa
LMX1A: LIM homeobox transcription factor 1α
MDS-UPDRS: Movement Disorder Society–Unified Parkinson’s Disease Rating Scale
Olig2: Oligodendrocyte transcription factor 2
PD: Parkinson’s disease
PET: Positron emission tomography
SHH: Sonic hedgehog
